# Influence of Phytase Supplementation at Increasing Doses from 0 to 1500 FTU/kg on Growth Performance, Nutrient Digestibility, and Bone Status in Grower–Finisher Pigs Fed Phosphorus-Deficient Diets

**DOI:** 10.3390/ani10050847

**Published:** 2020-05-14

**Authors:** Eugeniusz R. Grela, Siemowit Muszyński, Anna Czech, Janine Donaldson, Piotr Stanisławski, Małgorzata Kapica, Oksana Brezvyn, Viktor Muzyka, Ihor Kotsyumbas, Ewa Tomaszewska

**Affiliations:** 1 Institute of Animal Nutrition and Bromatology, Faculty of Animal Sciences and Bioeconomy, University of Life Sciences in Lublin, Akademicka St. 12, 20-950 Lublin, Poland; eugeniusz.grela@up.lublin.pl; 2 Department of Biophysics, Faculty of Environmental Biology, University of Life Sciences in Lublin, Akademicka St. 13, 20-950 Lublin, Poland; siemowit.muszynski@up.lublin.pl; 3 Department of Biochemistry and Toxicology, Faculty of Animal Sciences and Bioeconomy, University of Life Sciences in Lublin, Akademicka St. 12, 20-950 Lublin, Poland; anna.czech@up.lublin.pl; 4 School of Physiology, Faculty of Health Sciences, University of the Witwatersrand, 7 York Road, Parktown, Johannesburg 2193, South Africa; janine.donaldson@wits.ac.za; 5 DSM Nutritional Products Sp. z o.o., Tarczyńska 113, 96-320 Mszczonów, Poland; piotr.stanislawski@dsm.com; 6 Department of Animal Physiology, Faculty of Veterinary Medicine, University of Life Sciences in Lublin, Akademicka St. 12, 20-950 Lublin, Poland; malgorzata.kapica@up.lublin.pl; 7 State Scientific Research Control Institute of Veterinary Medicinal Products and Feed Additives, Donetska St. 11, 79000 Lviv, Ukraine; brezvun@gmail.com (O.B.); muzyka@scivp.lviv.ua (V.M.); dir@scivp.lviv.ua (I.K.)

**Keywords:** phytase, P and Ca digestibility, bone, mechanical testing, grower–finisher pigs

## Abstract

**Simple Summary:**

The current study investigates the growth performance and bone status of grower–finisher pigs supplemented with phytase, an enzyme which increases the bioavailability of phosphorus in animal feeds. The study results provide new information with regards to the positive role phytase supplementation to livestock feed plays in the achievement of the maximum effectiveness of the feed, as well as numerous positive effects on bone characteristics (geometry, mineralization, and mechanical strength) in grower–finisher pigs.

**Abstract:**

The objective of the current study is to assess the effects of the inclusion of 6-n phytase to a phosphorous-deficient diet on the growth performance (feed intake, average daily gain, and feed conversion ratio), apparent digestibility of calcium and phosphorus, and bone characteristics of grower–finisher pigs. The experimental diets included a phosphorus-deficient diet containing 0 (negative control), 250, 500, 1000, or 1500 FTU/kg of 6-phytase, and a diet formulated to meet the phosphorus nutrient requirements of pigs (positive control). Pigs were fed the experimental diets from the time they were ~35 kg body weight until they reached slaughter weight of ~110 kg. Bone status of the metacarpal (ash, mineral content) and femur (mineralization, geometry, and mechanical strength) bones were assessed. There was no effect of dietary treatment on feed intake. Feed conversion ratio was improved following inclusion of phytase at a dose of 500 FTU/kg or higher. Phytase inclusion at a dose of 1000 FTU/kg increased the average daily weight gain of grower–finisher pigs. Phytase inclusion at a dose of 500 FTU/kg was sufficient to increase metacarpal phosphorus content. Femur mid-diaphysis ash percentage was significantly increased even after the inclusion of the lowest dose of phytase. Analysis of structural parameters of femur mechanical strength (Young’s modulus, yield stress, yield strain, ultimate stress, ultimate strain) showed that the inclusion of a phytase dose of 500 FTU/kg in growing/finishing diets was sufficient to significantly improve bone status of grower–finisher pigs at slaughter.

## 1. Introduction

Over and above the involvement of phosphorous in the regulation of key metabolic enzymes and its action as an intracellular buffer for acid–base balance, phosphorous is also required for protein synthesis and cell metabolism, as well as for skeletal mineralization [1,2]. Phytate (inositol phosphate) is the primary storage compound of phosphorus in cereals and oilseeds seeds, which are commonly used in plant-based animal feeds. Phytases represent a subgroup of phosphomonoesterases that are capable of initiating the dephosphorylation of phytate. Due to the lack of significant endogenous phytase activity and a limited microbial population in the small intestine of monogastrics, their digestive tract has a very limited ability to hydrolyze phytate, making phytate-bound phosphorus virtually indigestible [3,4,5]. For this reason, the supplementation of phosphorus to feedstuff represents an appreciable cost factor in animal nutrition, with phosphorus being the third most expensive nutrient after energy and protein [1]. Additional phosphorous is commonly added to livestock feed in accordance with the recommendations, however the undigested phytate phosphorus is excreted and can contribute to environmental pollution, especially in areas with a high density of animal production [1].

However, the use of exogenous phytase improves bioavailability of phosphorus, calcium and iron as well as protein, amino acids, and other trace minerals and nutrients chelated with phytate for monogastric species such as poultry and swine [6]. Thus, dietary supplementation with exogenous phytase proves to be the most effective tool for livestock production in order to reduce phosphate excretion in animal waste, if phosphorus is minimal in the feed [7].

However, the efficacy of different phytases to release phosphorus differs between monogastric species. While fungal phytases have shown higher phosphorus release in pigs compared with broilers, *E. coli* phytase has shown a similar release of phosphorus across species. In swine, the phosphorus release generated by a 400 FTU/kg dose of phytase derived from *Peniophora lycii*, *A. niger*, and *E. coli* was 0.43, 0.81, and 1.08 g/kg P, respectively [8]. Furthermore, a 500 FTU/kg dose of phytase from *P. lycii* and *E. coli* generated a 0.572 and 0.770 g/kg release of phosphorus, respectively [9].

Bone as a metabolically active tissue is the main reservoir for phosphorus and calcium and participates in the maintenance of both phosphorus and calcium homeostasis [10]. Moreover, the quality of bones is a key factor influencing the maturity and functionality of the skeletal system, which provides support, locomotive functions, and counteracts the forces of gravity. Due to their supporting, carrying, and protective functions, bone tissue is constantly exposed to mechanical injuries caused by external forces [11]. Each injury of any element of the skeletal system poses a serious risk of economic losses [12]. Moreover, as the skeletal system is a place of attachment of muscles, disturbed bone metabolism by an insufficient supply of nutrients, including minerals or other disorders, can result in, among others, difficulties in movement and the changed behavior of the animal, leading to lowered feed intake [13]. Therefore, healthy bones allow the maintenance of mineral homeostasis, efficient movement, and proper behavior, all of which determine the general welfare of pigs.

Although a significant volume of literature has reported numerous effects of phytase on pig performance, to our knowledge, there are no reports on the effect of phytase supplementation on the biomechanical and geometric traits of bones from swine fed phosphorus-deficient diets. As bone quality depends on phosphorus and the development of the skeletal system at post-starter period influences the performance of pigs, as well as the risk of bone fractures during transport or procedures at slaughter, the objective of the current study is to evaluate the effects of dietary supplementation of *Aspergillus oryzae*-derived phytase at various doses ranging between 0–1500 FTU/kg on the body weight, nutrient digestibility and bone densitometry, and geometry and selected mechanical bone parameters of pigs fed a phosphorus-deficient diet during the grower/finisher stages of production.

## 2. Materials and Methods

The experimental procedures used throughout this study were approved by the II Local Ethics Committee on Animal Experimentation of University of Life Sciences in Lublin, Poland (resolution No. 6/2012).

### 2.1. Pigs

The current study made use of 432 Large White Polish x Polish Landrace crossbred pigs (216 barrows and 216 gilts). The pigs were managed according to normal commercial practice prior to the start of the trial and arrived at the grower/finisher unit weighing ~35 kg body weight (BW_st). The pigs were individually tagged, weighed, and allocated to one of six dietary treatment groups, with an equal number of barrows and gilts per group. Each dietary treatment consisted of eight pen replicates. Within a single replicate (pen), each treatment was balanced for pen size and sex distribution: 18 pigs (9 gilts + 9 barrows). Body weight variation within and between each pen replicate and group was minimized as far as it is practically possible. Pigs were housed in pens with straw for bedding during the experimental period.

### 2.2. Experimental Design

The treatments included a negative control (NC) group which received a phosphorus-deficient basal diet, experimental groups which received the NC diet supplemented with a 6-phytase produced by a genetically modified strain of *Aspergillus oryzae* (RONOZYME HiPhos with the declared minimal activity of 50,000 FTU/g, DSM Nutritional Products, Mszczonów, Poland) at doses of 250, 500, 1000 or 1500 FTU/kg feed, respectively [14], and a positive control (PC) group which received a diet formulated to meet the NCR nutrient requirements for grower–finisher pigs [15]. No other enzymes were added to the diets. Each treatment group was fed a different diet during the different growth stages, i.e., at 35–70 kg and 70–110 kg average BW (Table 1). The main ingredients of the NC and PC diets, which were prepared and pelleted by DSM Poland, were barley, wheat, maize, triticale, and soybean meal (Table 2). Pigs were fed ad libitum and had free access to water throughout the study period.

### 2.3. Feed Analysis

Feed samples were analyzed for basal nutrient content and for total P and Ca concentrations using standard AOAC methods [16] (Table 2). Crude protein, crude fiber, and crude fat in the diets were determined according to methods 954.01, 920.39 and 978.10, respectively. The total phosphorus was determined colorimetrically (method 965.17), whereas the Ca content was determined using the FAAS technique (method 968.08) [16].

The in-feed phytase activity was analyzed using a colorimetric enzymatic method (method 2000.12) [16]. One FTU is defined as the quantity of enzyme that can release about 1 μmol of inorganic phosphorus/minute from 5.0 mM sodium phytate at pH 5.5 at 37 °C [17]. Phytase activity in all diets was within the target dose range or slightly higher, as shown in Table 2.

### 2.4. Sampling and Measurements

Pigs were weighed every 14 days, and average daily weight gain (ADG) was calculated. Feed intake (FI) was recorded on a per pen basis and then transformed into an individual basis by dividing by the number of pigs per pen. The FI and feed conversion ratio (FCR) are expressed as a pen average for each 14-day period. Six weeks after the beginning of the trial, the pigs reached the target body weight of 60 kg (BW_gr) on average, when the finisher diet was introduced (Table 1). When pigs reached the average target weight of 110 kg for the pen (BW_fin), they were sent for slaughter. Results for ADG, FI, and FCR were calculated for the starter (BW_st—BW_gr) and grower (BW_gr—BW_fin) periods, as well as the mean for the whole fattening period (BW_st—BW_fin).

During the digestibility study periods (grower: 45–50 kg BW; finisher: 105–110 kg BW), the feed mixtures for all groups were enriched before granulation with 3 g Cr_2_O_3_ per kg as a digestibility marker [18]. Fecal samples were collected for two consecutive days from each treatment pen replicate and were pooled by pen, labeled, and stored at −20 °C for subsequent analysis.

The chromic oxide in feed mixtures and fecal samples was determined according to the method described by Suzuki and Early [18]. Following thawing and homogenization, fecal samples were analyzed for DM, organic matter, and Ca and P content, using standard AOAC methods [16]. Apparent digestibility coefficients (*ADC*) of Ca and P were calculated using the following formula:

$$ADC\;\left(\%\right)=100\left(1-\frac{Ni\cdot Md}{Nd\cdot Mi}\right)$$

where *Ni* represents the concentration of the nutrient (Ca or P) in the feces, *Md* represents the dietary concentration of the marker (chromic oxide), *Nd* represents the dietary concentration of the nutrient (Ca or P), and *Mi* represents the concentration of the marker in the feces (all values expressed in g/kg DM) [19]. Ca and P intake was calculated using the daily FI multiplied by the dietary Ca and P levels for each treatment and growth period (grower, finisher). Organic matter digestibility coefficient (OMDC) was calculated using the equations described by Adeola [20].

After final weighing (~110 kg) at slaughter, *n* = 8 barrows from each experimental group were selected (i.e., one barrow from each pen with a body weight closest to the average body weight of the group, 48 pigs in total) for bone analysis. Only barrows were used to avoid differences that might exist between sexes in the bone traits measured, since the sex hormones may have an effect on bone mineralization [21,22,23,24,25,26,27]. The right femur and third metacarpal bone were dissected out, cleaned from adherent tissues, wrapped in gauze soaked in 0.9% saline, and frozen at −20 °C until further analyses.

### 2.5. Bone Analysis

The defatted metacarpal bone was dried at 105 °C for 16 h to determine bone DM weight. Next, the bone was ashed in a muffle furnace at 650 °C for 24 h to determine the bone ash percentage. Finally, the obtained ash was dissolved in aqua regia (3 parts HCl + 1 part HNO_3_) and analyzed for Ca and P content using the standard AOAC methods described above for feed samples. Ash percentage, and Ca and P content, were expressed relative to dry bone weight.

Before analyses, the frozen femora were thawed overnight in the laboratory at 10 °C. Measurement of whole bone mineral density (BMD) and bone mineral content (BMC) was performed using the dual-energy X-ray absorptiometry (DXA) method on a Discovery W densitometer (Hologic Inc., Bedford, MA, USA). To examine the mechanical properties of the femora, a three-point bending test was performed on a universal testing machine (Zwick Z010, Zwick-Roell GmbH & Co., Ulm, Germany). The load was applied to the midpoint of the bone diaphysis until fracture. A fixed span of 75 mm (about 40% of total bone length) and a constant load rate of 10 mm/min were used. Next, the femora were cut across in the midpoint of the bone diaphysis with a diamond bandsaw (MBS 240/E, Proxxon GmbH, Foehren, Germany) and bone diaphysis geometric properties (cortical index, cross-sectional area, and cross-sectional moment of inertia) were determined on the basis of measurements of cortical bone cross-sectional diameters (internal and external), using a digital caliper [28]. On the basis of recorded load–deformation curves and measured diameters, bone mechanical (yield load, fracture load, stiffness, and stiffness) and material properties (Young’s modulus, yield strain, yield stress, fracture strain, and fracture stress) of the femora were determined using standard engineering beam-theory equations, using Origin software (OriginLab, Northampton, MA, USA) as previously described [29]. Once the geometrical measurements were complete, the femora mid-diaphysis were cut off. After washing, they were defatted using chloroform–methanol (2:1) at 37 °C for 24 h and dried at 105 °C to a constant weight to determine DM. Finally, bone fragments were mineralized in a muffle furnace at 650 °C for 24 h, and crude ash percentage was calculated.

### 2.6. Statistical Analysis

All data are expressed as means and SEM (standard error of the means). Data were analyzed using Statistica 13 (Dell Software Inc., USA). A one-way ANOVA was used to assess parametric data with treatment as the fixed effect and pen (FI, ADG, FCR, ADC analysis) or pig (bones analysis) as the experimental unit. Treatment means were compared using Tukey’s HSD (honest significant difference) test. The normality of data distribution was tested using the Shapiro–Wilk test, and equality of variance was tested by Levene’s test. Orthogonal polynomial contrasts were performed on the 5 phosphorus-deficient treatments (NC, NC + 250 FTU/kg, NC + 500 FTU/kg, NC + 1000 FTU/kg, and NC + 1500 FTU/kg) to test the linear and quadratic effects of phytase levels on selected response variables. For all tests, a criterion α level of *p* < 0.05 was used to determine statistical significance.

## 3. Results

Performance results are presented in Table 3. The pigs fed the NC diet showed lower ADG compared to those fed the PC diet during the grower period, and ADG increased with phytase level (linear, *p* < 0.01). ADG for the finisher period also showed a linear increase (*p* < 0.05). Mean ADG, during the whole fattening period, in the group of pigs fed the NC diet was lower compared to the PC diet. However, increasing phytase dose increased mean ADG (linear and quadratic, *p* < 0.05 in both cases). During both the grower and finisher periods, the pigs fed the phosphorus-deficient diet supplemented with phytase at a dose of 1000 FTU/kg showed higher ADG compared to those fed the NC diet.

Pigs fed the NC diet showed increased FCR during the grower period than those fed the PC diet, and FCR decreased with increasing phytase level in phosphorus-deficient diets (linear and quadratic, *p* < 0.001 and *p* < 0.05, respectively). During the finisher period, FCR also decreased with increasing phytase level (linear and quadratic, *p* < 0.05 for both cases), but for all pigs fed the phosphorus-deficient diets, FCR was not different from that observed for pigs fed the PC diet. During the whole fattening period, pigs fed the NC diet showed increased mean FCR compared to those fed the PC diet. There was also a significant effect of phytase level on mean FCR (linear and quadratic, *p* < 0.001 and *p* < 0.01, respectively). Pigs receiving the NC diet supplemented with phytase at a dose of 500 or 1000 FTU/kg showed lower mean FCR values when compared to those receiving only the NC diet.

The effects of inclusion of increasing phytase doses to phosphorus-deficient diets on organic matter digestibility coefficient (OMDC) and apparent digestibility coefficients (ADC) of calcium and phosphorus are presented in Table 4. There was no effect of dietary treatment on OMDC in both fattening periods. During the grower period, calcium ADC increased with increasing phytase level (linear, *p* < 0.001). However, pigs fed the NC diet and the diet supplemented with 250 or 500 FTU/kg showed significantly lower calcium ADC compared to the PC diet. During the finisher period, pigs fed the NC diet had significantly lower calcium ADC when compared to those fed the finisher PC diet; however, calcium ADC increased with increasing phytase level, showing both a linear and quadratic response (*p* < 0.001 and *p* < 0.05, respectively). Mean calcium ADC in pigs fed the NC diet was significantly lower when compared to that of pigs fed the PC diet; however, it showed a linear (*p* < 0.001) effect with increasing levels of phytase.

During the whole fattening period (grower and finisher periods, as well as mean ADC) the ADC of phosphorus was significantly increased (*p* < 0.001) in pigs fed diets supplemented with phytase, irrespective of the dose, compared with that of the NC group, and showed both linear and quadratic effects with increasing levels of phytase (*p* < 0.001 for both effects). Moreover, pigs fed the NC diet and the diet supplemented with 250 FTU/kg of phytase showed a significant decrease in phosphorus ADC compared to that of pigs fed the PC diet during the whole fattening period (grower, finisher, and mean). On the other hand, pigs fed phosphorus-deficient diets supplemented with 1000 or 1500 FTU/kg of phytase showed an increase in the ADC of phosphorus during the whole fattening period when compared to that of pigs fed the PC diet.

Bone ash percentage, calcium, and phosphorus content, as well as the Ca:P ratio in metacarpal bone in pigs at slaughter, are presented in Table 5. Contrast analysis indicated that Ca content increased with increasing phytase level in the phosphorus-deficient diets (linear, *p* < 0.01). Phosphorus content in metacarpal bone was significantly lower (by 5.1%) in pigs fed the NC diet compared to those fed the PC diet. However, it increased linearly in pigs fed phosphorus-deficient diets supplemented with phytase (*p* < 0.001). The Ca:P ratio decreased linearly (*p* < 0.01) in pigs fed phosphorus-deficient diets supplemented with phytase. Pigs fed the diet supplemented with 1000 FTU/kg of phytase had a significantly lower bone Ca:P ratio compared to that of the NC group.

The general and geometric properties of the femora are presented in Table 6. The weight of femora from pigs fed phosphorus-deficient diets increased with increasing phytase levels (linear and quadric, *p* < 0.001 for both effects). Femora from pigs fed the phosphorus-deficient diet supplemented with phytase at a dose of 1500 FTU/kg were heavier than those from pigs fed only the NC diet and those fed the PC diet (*p* < 0.001, Table 6). Femora from pigs fed the phosphorus-deficient diet supplemented with phytase at a dose of 1500 FTU/kg were also longer than those from pigs fed the PC diet (*p* < 0.05, Table 6). There was also an effect of dietary treatment on the femur cortical index (*p* < 0.001). The cortical index of femora from pigs fed a phosphorus-deficient diet supplemented with phytase at a dose of 1000 FTU/kg was significantly lower when compared to that from pigs fed the NC diet and the PC diet, while for pigs supplemented with phytase at a dose of 1500 FTU/kg, the cortical index was lower when compared to that from pigs fed the PC diet only and did not differ from that of pigs from the NC group. In pigs fed phosphorus-deficient diets, the femora mid-diaphysis cross-sectional area (CSA) decreased with increasing phytase levels (linear, *p* < 0.001). However, only pigs supplemented with phytase at a dose of 1000 FTU/kg had a significantly lower femur CSA compared to those of the PC group. Femora cross-sectional moment of inertia (CSMI) also increased with increasing phytase levels, showing both a linear and quadratic response (*p* < 0.01 for both effects). Pigs fed phosphorus-deficient diets supplemented with 250 or 500 FTU/kg phytase had increased femora CSMI compared to pigs fed both the NC and the PC diets. Ash content of the femoral diaphysis increased with increasing phytase levels (linear and quadratic, *p* < 0.001 for both effects). Femoral mid-diaphysis ash content was significantly lower in pigs fed the NC diet compared to that observed in all other groups, including pigs fed the PC diet. Similar femoral mid-diaphysis BMD and BMC (*p* > 0.05) were observed across groups at slaughter.

Mechanical properties of femora from pigs at slaughter are presented in Table 7. While there was a quadratic increase in femoral stiffness (*p* < 0.01), the femora yield force was not different between groups (*p* > 0.05). Pigs fed the P-deficient diet supplemented with phytase at a dose of 500 FTU/kg showed lower breaking force compared to those fed the PC and those fed the NC diet. Femora Young’s modulus increased with increasing phytase level (linear, *p* < 0.001), peaking in pigs fed a phosphorus-deficient diet supplemented with 1000 and 1500 FTU/kg phytase. Femora Young’s modulus was significantly lower in pigs fed the phosphorus-deficient control NC diet and those fed the phosphorus-deficient diet supplemented with 250 FTU/kg phytase compared to pigs fed the PC diet. Increasing phytase dose decreased yield strain (linear and quadratic, *p* < 0.001 for both effects). Among all dietary treatments, the greatest elastic deformations (in terms of yield strain) were observed for pigs fed the NC diet, and those fed the phosphorus-deficient diet supplemented with 250 FTU/kg phytase. A linear decrease in yield stress in pigs fed the phosphorus-deficient diets supplemented with phytase (*p* < 0.5) was observed, with yield stress being significantly lower in pigs fed the phosphorus-deficient diet supplemented with either 250 or 500 FTU/kg phytase compared to that observed in pigs supplemented with a higher dose of phytase (1000 FTU/kg). A linear decrease in breaking strain was observed with increasing phytase level (linear, *p* < 0.001), with the highest breaking strain values observed for pigs fed the NC (0 FTU/kg) diet, and those fed the P-deficient diet supplemented with the lowest dose of phytase (250 FTU/kg). The breaking strain of femora in these groups was also significantly increased when compared to the group fed the PC diet. Femora breaking stress was significantly affected by dietary treatment (*p* < 0.01). For the phosphorus-deficient diets, this effect was linear (*p* < 0.05). Femora breaking stress was significantly lower in pigs from the NC group, and those from the groups supplemented with 250 or 500 FTU/kg phytase, compared to that of pigs from the group supplemented with phytase at a dose of 1000 FTU/kg and the PC group.

## 4. Discussion

Bones, which form part of the highly specialized weight-supporting framework of the body, must endure voluntary physical activities without breaking or causing pain. Bone should be strong enough to maintain its load-bearing capacity to allow animals to keep their behavior. Factors which influence the load-bearing capacity of bone can be divided into mechanical factors, which are associated with body weight, and non-mechanical factors, including genetics, gender, hormones, vitamins (mainly vit. D), cytokines and minerals [30]. These non-mechanical factors directly affect body weight and thus indirectly influence the load placed on bone. Non-mechanical factors can augment or weaken mechanical properties of bone [30,31]. Previous studies have demonstrated beneficial effects of increasing doses of phytase on growth performance and bone strength in pigs [32,33,34,35,36], however comprehensive research into the effects of different phytase doses on the densitometry, geometric, structural and material properties of femora in grower–finisher pigs fed phosphorus-deficient diets is lacking.

In our study, ADG and FCR were influenced by dietary treatment, while FI was not affected. The unchanged FI was observed in study involving pigs supplemented with phytase at the dose of 500 FTU/kg described by Schlegel and Gutzwiller [35]. In other study in which weaned piglets were administered various doses of phytase (500, 1000, or 2000 FTU/kg) for a period of 14 days, a higher ADG and FI was observed in piglets administered phytase at a dose of 2000 FTU/kg [34]. Santos et al. [22] observed a significantly higher ADG, following supplementation of phytase at a dose of 2000 FTU/kg to growing pigs (between 23 and 55 kg body weight), compared to those receiving phytase at a dose of 250 or 500 FTU/kg, together with a calcium- and phosphorus-deficient diet, despite all dietary groups having a similar FI [22]. Feed intake was also unchanged in a study by Tsai et al. [37] performed on 20-kg barrows fed a diet with low or adequate phosphorus supplemented with phytase at doses of 250, 500, 2500 or 12,500 FTU/kg, for a period of 14 days; however, the highest ADG was observed in the barrows fed the diet supplemented with phytase at a dose of 2500 FTU/kg [37]. In another study, Varley et al. [38] showed no significant differences in FI, ADG, or FCR in weaned piglets with a body weight of approximately 33 kg, fed a diet with an adequate amount of P supplemented with phytase at a dose of 500, 1000, or 1500 FTU/kg, during the weaner and grower–finisher periods [38]. In contrast, in the recent study Wiśniewska et al. [39] showed that the phytase supplemented to the calcium- and phosphorus-deficient diet at a dose of 250 FTU/kg for a period of 42 days significantly improved ADG and FCR, as well as ADC for Ca and P compared to the control calcium- and phosphorus-deficient diet [39]. ADC for phosphorus availability in a study by Dersjant-Lia et al. was significantly higher, but only compared with the phosphorus-deficient diet, and increasing the phytase dose had no effect [34]. Opposing results have been observed by Tsai et al. [37] in barrows fed diets with low or adequate phosphorus supplemented with phytase at a dose of 250, 500, 2500 or 12,500 FTU/kg, for a period of 14 days. The ADC for phosphorus was the highest in the barrows fed the phosphorus-deficient diet supplemented with phytase at a dose 2500 or 12,500 FTU/kg, while for a phosphorus-adequate diet, a phytase at the lowest dose of 250 FTU/kg was sufficient to increase the ADC for phosphorus to the same extent as observed for barrows supplemented with higher doses of phytase [37].

The change in ADC for phosphorus and calcium can influence the mineral composition of the bones, as evidenced in the present study by the changes in mineralization observed within the metacarpal bone. It should be noted that the ash content of the metacarpal bone was not significantly altered despite the changes in Ca:P ratio induced by the changes in phosphorus content. On the other hand, the densitometric analysis of the femora showed no difference between treatment groups in our study. Femur ash content was determined only for cleaned and defatted bone mid-diaphysis, while densitometry was performed for the whole femur bone. Thus, the femur ash results could suggest that significant changes in mineralization only occurred in the bone diaphysis region. Our earlier studies have showed that differences in BMD and BMC exist in different parts of the bone (distal and proximal epiphysis or bone mid-diaphysis) [29,40,41,42]. A previous study showed that phytase supplementation at increasing doses ranging from 0 (NC) to 1500 FTU/kg to a phosphorus-deficient diet significantly increased femur ash content in young growing pigs [36]. Similarly, the phytase added at the dose of 500 FTU/kg to a calcium- and phosphorus-deficient diet increased BMD of metacarpal bone in pigs at slaughter [43]. There are, however, other studies that observed no effects of phytase supplementation on bone mineral content (BMC) or bone ash of metacarpal bones, but these studies made use of calcium- and phosphorus-deficient diets supplemented with phytase at a dose of 500 or a dose of 250 FTU/kg [39,44]. It can be due to fact that in different bones, changes in ash content, BMC, and BMD are observed due to their different phosphorus supply [45,46]. On the other hand, all investigated bone types react similarly to the dietary stimulus. For decades, metacarpal bone, due to its structure, has been used as a suitable model bone for the diagnosis of mineral deficiencies in pigs [21,47]. Santos et al. [22] indicated that the bone-breaking strength of metacarpal bone is reduced in pigs fed calcium and phosphorus-deficient diets and supplementation with phytase at a dose of 500 FTU/kg is sufficient to improve the results of mechanical testing. Further increases in phytase dose did not show any additional improvements in bone mechanical properties and the authors attributed this lack of further improvement to possible phytase destruction rather than the provision of phosphorus [22]. However, they do not describe how the bone-breaking strength is determined in their study. In another study, where the metacarpal bones were subjected to three-point bending with loading rate of 2 mm/s, an increase in breaking force in pigs fed a diet with the recommend phosphorus content supplemented with phytase at a dose of 500 FTU/kg was observed [35]. A further increase of breaking strength was observed when the same dose of phytase is added to the diet with increasing Ca:digestible P ratio, with no changes in growth performance [35].

It seems that metacarpal bone is more suitable for routine examinations because its extraction at slaughter is easier than that of the larger bones, such as the femur [45]. However, the femur is considered a model bone for mechanical testing in quadrupedal animals, with the three-point bending test being the most suitable, since the femur generally sustains bending stress [11,48,49,50,51], as metacarpal and femur bones are subjected to different stresses in the living animal due to their location in the body [48]. To our knowledge, our study is the first to show the effects of phytase supplementation on numerous geometric and mechanical characteristics of the femur. These results indicate that the P-deficient diet had no influence on femur breaking force or stiffness. However, bone material properties, like Young’s modulus, stress, and strain, are better traits than raw bone breaking strength or stiffness in measuring the effect of dietary treatment on bone mechanical strength since they correct for bone length and structural organization, as well as testing procedure (loading rate, span distance) [23,29,52,53,54]. Additionally, bone mid-diaphysis geometric indices also provide important information concerning the strength of long bones since, during bending, the bone’s internal resistance to an imposed load (stress) is directly dependent on the spatial distribution of bone mass [53,55,56]. The increased femur yield and breaking strains observed in the pigs fed the NC diet and in those fed the phosphorus-deficient diet supplemented with phytase at a dose of 250 FTU/kg indicate that the bones of these pigs showed a greater bone deflection in both the elastic and plastic regions. This rubber-like nature of the bones was also proved by the lowest Young‘s modulus being observed in the above-mentioned groups. This is at least partially due to their reduced mineralization of the bone mid-diaphysis, as shown by the lowered ash content. The aforementioned results all suggest that the femora from pigs in the NC and 250 FTU/kg groups were significantly less rigid and more prone to deformation compared to those from pigs in the remaining dietary groups.

When bone is subjected to bending load, lower stresses for a bone with greater cross-sectional moment of inertia will be observed and whole bone strength depends on the spatial distribution of bone mass in the mid-diaphysis of the bone which bears the load [55,57]. The calculated bending yield and ultimate stress in femora of pigs from the 500 FTU/kg group, which was characterized by the greatest CSMI, were lower than those observed for the 1000 FTU/kg and PC groups, in which CSMI were significantly lower. This may be the reason for differences in the yield load observed between the 500 FTU/kg group and 1000 FTU/kg group or the PC group. However, the bone tendency to deformation and fracture under the action of external forces may also be associated with the structure of the organic phase. Therefore, in order to be able to explain the other differences observed in the current study, an analysis of the organic phase of bone is probably required, especially with regards to the collagen network, which is responsible not only for the elastic properties of bone, but also contributes to overall bone integrity, since it provides a structural scaffolding to the inorganic, mineral phase. In our study, a further increase in phytase dose (1500 FTU/kg) resulted in the highest bone weight and length without any significant influence on the geometric, mechanical, and material bone parameters. Only one previous study, to our knowledge, has investigated and reported on bone geometric parameters, but their data relate only to metacarpal bone and indicate that phytase supplementation at a dose of 500 FTU/kg does not influence metacarpal cross-sectional area or cross-sectional moment of inertia [43].

## 5. Conclusions

In conclusion, the feed conversion ratio was improved following the inclusion of phytase at a dose of 500 FTU/kg or higher, while phytase inclusion at a dose of 1000 FTU/kg increased the average daily weight gain of grower–finisher pigs. Results of the present study show that the 6-phytase dose of 500 FTU/kg seems to be adequate for economic cost in fattening. As expected, supplementing increasing levels of phytase in phosphorus-deficient diets resulted in improvements in bone parameters. For the metacarpal, phytase inclusion at a dose of 500 FTU/kg was sufficient to increase bone phosphorus content. Analysis of structural parameters of femur mechanical strength showed that the inclusion of a phytase dose of 500 FTU/kg in growing/finishing diets was sufficient to significantly improve the bone status of grower–finisher pigs at slaughter.

## Figures and Tables

**Table 1:** Ingredients of basal grower and finisher diets.

Ingredient (%)	Grower (~35–70 kg)		Finisher (~70–110 kg)	
	NC	PC	NC	PC
Barley	34.98	35.09	39.94	40.07
Wheat	29.00	28.00	28.60	27.50
Triticale	15.00	15.00	15.00	15.00
Soybean meal, 45% CP	16.90	17.10	13.70	13.90
Di-calcium phosphate	0.50	1.05	0.25	0.80
Limestone	0.58	0.52	0.63	0.56
Methionine	0.05	0.05	0.04	0.04
L-lysine HCl	0.32	0.32	0.28	0.27
Sodium chloride	0.47	0.47	0.47	0.47
Soybean oil	1.60	1.80	0.50	0.80
L-threonine	0.10	0.10	0.09	0.09
Mineral-vitamin premix ^*^	0.50	0.50	0.50	0.50
Total	100.00	100.00	100.00	100.00

NC–the negative control basal diet; PC–the positive control basal diet * Mineral–vitamin premix, content in 1 kg: Ca 257.93 g, Mg 2.20 g, K 0.209 g, Na 0.035 g, Fe 20 g, Mn 10 g, Zn 10 g, Cu 2,5 g, Se 40.0 mg, J 100 mg, Co 80 mg, vit. A 1,600,000 IU, vit. D3 200,000 IU, vit. E 10.0 g, vit. K 300 mg, vit. B1 200 mg, vit. B2 600 mg, vit. B6 300 mg, vit. B12 5.0 mg, nicotic acid 4.0 g, pantothenic acid 2.4 g, chloric choline 40 g, folic acid 40 mg, antioxidant (butylated hydroxyanisole, BHA, E320) 120 mg. NC–the negative control basal diet; PC–the positive control basal diet.

**Table 2:** Basal nutrient content, total P and Ca concentrations (g/kg), and total phytase activity (FTU/kg) in grower and finisher diets for different treatment groups.

Treatment ^1^	NC	NC + 250	NC + 500	NC + 1000	NC + 1500	PC
**Grower**						
Dry matter	876.5	873.0	879.8	880.3	882.3	87.93
Crude ash	41.1	41.2	41.5	39.8	40.2	41.1
Crude protein	180.4	177.6	179.9	179.5	179.7	180.1
Ether extract	34.2	33.4	32.6	29.8	32.8	30.5
Crude fibre	17.2	18.0	16.3	15.7	15.8	15.8
Nitrogen free extract	603.6	602.8	609.5	615.5	613.8	611.8
Calcium	5.51	5.45	5.47	5.49	5.57	6.62
Total phosphorus	4.82	4.74	4.75	4.78	4.83	5.69
Phytase activity (FTU/kg)	187	411	639	1081	1574	195
**Finisher**						
Dry matter	871.6	867.7	869.2	871.3	868.9	869.7
Crude ash	42.6	43.2	42.8	43.9	40.1	43.6
Crude protein	163.2	162.5	162.6	161.3	161.1	162.8
Ether extract	28.9	25.0	21.0	23.8	22.5	22.3
Crude fibre	19.9	19.7	20.2	21.2	21.9	21.6
Nitrogen free extract	617.0	617.3	622.6	621.1	623.3	619.4
Calcium	4.94	4.92	4.98	4.95	4.94	6.09
Total phosphorus	4.25	4.23	4.29	4.27	4.31	5.27
Phytase activity (FTU/kg)	209	394	699	1160	1577	205

^1^ There were a total of 6 dietary treatments. Diets 1–5 were phosphorus-deficient diets: with 0 (NC), 250, 500, 1000, or 1500 phytase units FTU/kg (RONOZYME HiPhos phytase, DSM, Poland), respectively. A sixth diet (positive control; PC) was formulated with increased di-calcium phosphate to meet the nutrient requirements of the pigs [15].

**Table 3:** Feed intake (FI), average daily gain (ADG), and feed conversion ratio (FCR) of pigs during the grower, finisher, and whole fattening periods.

Treatment ^1^	FI Grower, kg	FI Finisher, kg	FI Mean, kg	ADG Grower, kg/Day	ADG Finisher, kg/Day	ADG Mean, kg/Day	FCR Grower, kg/kg	FCR Finisher, kg/kg	FCR Mean, kg/kg
NC ^2^	1.98	2.61	2.35	0.789 ^a^	0.835 ^a^	0.815 ^a^	2.51 ^b^	3.12 ^b^	2.85 ^b^
250 ^3^	1.99	2.40	2.24	0.820 ^ab^	0.846 ^ab^	0.836 ^ab^	2.42 ^ab^	3.02 ^ab^	2.75 ^ab^
500 ^3^	1.97	2.55	2.31	0.851 ^ab^	0.845 ^ab^	0.849 ^ab^	2.31 ^a^	2.98 ^ab^	2.68 ^a^
1000 ^3^	1.96	2.54	2.30	0.864 ^b^	0.874 ^b^	0.870 ^b^	2.27 ^a^	2.89 ^a^	2.60 ^a^
1500 ^3^	1.98	2.56	2.37	0.849 ^ab^	0.849 ^ab^	0.849 ^ab^	2.32 ^a^	3.00 ^ab^	2.77 ^ab^
PC ^4^	1.96	2.55	2.31	0.862 ^b^	0.852 ^ab^	0.856 ^b^	2.27 ^a^	2.99 ^ab^	2.68 ^a^
SEM ^5^	0.16	0.14	0.09	0.046	0.023	0.024	0.12	0.14	0.10
*p*-value									
TRT ^6^	0.999	0.116	0.276	0.015	0.044	0.001	<0.001	0.052	<0.001
PHY ^7^	0.999	0.120	0.232	0.033	0.022	0.003	0.001	0.033	<0.001
Linear ^8^	0.929	0.752	0.885	0.005	0.035	0.029	<0.001	0.024	<0.001
Quadratic ^8^	0.959	0.149	0.116	0.106	0.175	0.032	0.030	0.047	0.004

FI = feed intake; ADG = average daily gain; FCR = feed conversion ratio. ^a,b^ Means with different superscripts, within the same column, are statistically different from one another (*p* < 0.05) based on Tukey’s post hoc test. ^1^ There were a total of 6 dietary treatments. Diets 1–5 were phosphorus-deficient diets: with 0 (NC), 250, 500, 1000, or 1500 phytase units FTU/kg (RONOZYME HiPhos phytase, DSM, Poland), respectively. A sixth diet (positive control; PC) was formulated with increased di-calcium phosphate to meet the nutrient requirements of the pigs [15]. ^2^ NC—negative control. ^3^ Phytase dose (FTU/kg feed) added to NC. ^4^ PC—positive control. ^5^ SEM—standard error of the mean. ^6^ *p*-value for overall effect of dietary treatment (Diets 1–6). ^7^ *p*-value for phytase effect in phosphorus-deficient diets (Diets 1–5). ^8^ Orthogonal polynomial (linear and quadratic) contrasts were performed to test the effect of phytase levels in the phosphorus-deficient diets.

**Table 4:** Organic matter digestibility coefficient (OMDC, %) and apparent digestibility coefficients (ADC, %) of Ca and P of pigs during the grower, finisher, and whole fattening periods.

Treatment ^1^	OMDC Grower	OMDC Finisher	Ca Grower	Ca Finisher	Ca Mean	P Grower	P Finisher	P Mean
NC ^2^	84.1	85.0	52.8 ^a^	62.9 ^a^	57.8 ^a^	39.4 ^a^	49.1 ^a^	44.2 ^a^
250 ^3^	84.2	85.2	53.8 ^ab^	64.2 ^ab^	59.0 ^ab^	46.3 ^b^	56.2 ^b^	51.2 ^b^
500 ^3^	84.5	85.7	53.4 ^ab^	64.7 ^b^	59.1 ^ab^	50.1 ^cd^	62.4 ^c^	56.3 ^c^
1000 ^3^	84.7	85.7	55.1 ^bc^	65.1 ^b^	60.1 ^b^	52.7 ^de^	66.9 ^d^	59.7 ^d^
1500 ^3^	84.7	85.6	55.1 ^bc^	64.9 ^b^	60.0 ^b^	54.7 ^e^	68.1 ^d^	61.4 ^d^
PC ^4^	84.3	85.4	55.6 ^c^	64.5 ^b^	60.1 ^b^	48.5 ^c^	64.4 ^c^	56.5 ^c^
SEM ^5^	0.5	0.6	1.2	0.9	0.8	1.7	1.5	1.2
*p*-value								
TRT ^6^	0.156	0.211	<0.001	<0.001	<0.001	<0.001	<0.001	<0.001
PHY ^7^	0.141	0.123	0.003	<0.001	<0.001	<0.001	<0.001	<0.001
Linear ^8^	0.012	0.020	<0.001	<0.001	<0.001	<0.001	<0.001	<0.001
Quadratic ^8^	0.769	0.296	0.096	0.011	0.187	<0.001	<0.001	<0.001

^a–e^ Means with different superscripts, within the same column, are statistically different from one another (*p* < 0.05) based on Tukey’s post hoc test. ^1^ There were a total of 6 dietary treatments. Diets 1–5 were phosphorus-deficient diets: with 0 (NC), 250, 500, 1000, or 1500 phytase units FTU/kg (RONOZYME HiPhos phytase, DSM, Poland), respectively. A sixth diet (positive control; PC) was formulated with increased di-calcium phosphate to meet the nutrient requirements of the pigs [15]. ^2^ NC—negative control. ^3^ Phytase dose (FTU/kg feed) added to NC. ^4^ PC—positive control. ^5^ SEM—standard error of the means. ^6^ *p*-value for overall effect of dietary treatment (Diets 1–6). ^7^ P-value for phytase effect in phosphorus-deficient diets (Diets 1–5). ^8^ Orthogonal polynomial (linear and quadratic) contrasts were performed to test the effect of phytase levels in the phosphorus-deficient diets.

**Table 5:** Crude ash and mineral content (g/kg DM) of metacarpal bone from grower–finisher pigs slaughtered at 110 kg BW.

Treatment ^1^	Ash, %	Ca, g/kg	P, g/kg	Ca:P
NC ^2^	42.8	168	72.0 ^a^	2.34 ^b^
250 ^3^	43.8	170	73.7 ^ab^	2.30 ^ab^
500 ^3^	43.7	171	75.0 ^bc^	2.29 ^ab^
1000 ^3^	44.0	172	77.2 ^c^	2.23 ^a^
1500 ^3^	44.0	171	76.6 ^c^	2.24 ^ab^
PC ^4^	43.7	172	75.9 ^bc^	2.26 ^ab^
SEM ^5^	1.1	3	1.6	0.07
*p*-value				
TRT ^6^	0.314	0.048	<0.001	0.024
PHY ^7^	0.227	0.038	<0.001	0.028
Linear ^8^	0.062	0.005	<0.001	0.002
Quadratic ^8^	0.319	0.135	0.096	0.623

Ca:P = bone Ca to P ratio. ^a–c^ Means with different superscripts, within the same column, are statistically different from one another (*p* < 0.05) based on Tukey’s post hoc test. ^1^ There were a total of 6 dietary treatments. Diets 1–5 were phosphorus-deficient diets: with 0 (NC), 250, 500, 1000, or 1500 phytase units FTU/kg (RONOZYME HiPhos phytase, DSM, Poland), respectively. A sixth diet (positive control; PC) was formulated with increased di-calcium phosphate to meet the nutrient requirements of the pigs [15]. ^2^ NC—negative control. ^3^ Phytase dose (FTU/kg feed) added to NC. ^4^ PC—positive control. ^5^ SEM—standard error of the means. ^6^ *p*-value for overall effect of dietary treatment (Diets 1–6). ^7^ *p*-value for phytase effect in phosphorus-deficient diets (Diets 1–5). ^8^ Orthogonal polynomial (linear and quadratic) contrasts were performed to test the effect of phytase levels in the phosphorus-deficient diets.

**Table 6:** General and geometric properties of femora of grower–finisher pigs slaughtered at 110 kg BW.

Treatment ^1^	Weight, g	Length, cm	Cortical index, %	CSA, mm^2^	CSMI, cm^4^	BMD, g/cm^2^	BMC, g	Ash, %
NC ^2^	261 ^a^	18.4 ^ab^	41.0 ^bc^	295 ^bc^	1.38 ^a^	1.05	73.5	64.8 ^a^
250 ^3^	276 ^ab^	18.5 ^ab^	40.2 ^abc^	321 ^c^	1.81 ^b^	1.07	79.2	66.6 ^b^
500 ^3^	263 ^a^	18.5 ^ab^	41.7 ^bc^	326 ^c^	1.86 ^b^	1.09	78.3	68.2 ^c^
1000 ^3^	265 ^a^	18.8 ^ab^	33.5 ^a^	230 ^a^	1.04 ^a^	1.19	85.4	68.4 ^c^
1500 ^3^	306 ^b^	19.2 ^b^	39.2 ^ab^	264 ^ab^	1.21 ^a^	1.14	90.5	67.5 ^bc^
PC ^4^	259 ^a^	18.3 ^a^	46.9 ^c^	296 ^bc^	1.23 ^a^	1.14	80.8	67.0 ^bc^
SEM ^5^	23	5.6	4.7	35.9	0.36	0.20	16.2	1.0
*p*-value								
TRT ^6^	<0.001	0.027	<0.001	<0.001	<0.001	0.773	0.387	<0.001
PHY ^7^	<0.001	0.013	0.011	<0.001	<0.001	0.716	0.288	<0.001
Linear ^8^	0.001	0.001	0.060	<0.001	0.007	0.223	0.094	<0.001
Quadratic ^8^	0.001	0.151	0.576	0.061	0.005	0.862	0.754	<0.001

CSA = cross-sectional area; CSMI = cross-sectional moment of inertia; BMD = bone mineral density; BMC = bone mineral content. ^a–c^ Means with different superscripts, within the same column, are statistically different from one another (*p* < 0.05) based on Tukey’s post hoc test. ^1^ There were a total of 6 dietary treatments. Diets 1–5 were phosphorus-deficient diets: with 0 (NC), 250, 500, 1000, or 1500 phytase units FTU/kg (RONOZYME HiPhos phytase, DSM, Poland), respectively. A sixth diet (positive control; PC) was formulated with increased di-calcium phosphate to meet the nutrient requirements of the pigs [15]. ^2^ NC—negative control. ^3^ Phytase dose (FTU/kg feed) added to NC. ^4^ PC—positive control. ^5^ SEM—standard error of the means. ^6^ *p*-value for overall effect of dietary treatment (Diets 1–6). ^7^ P-value for phytase effect in phosphorus-deficient diets (Diets 1–5). ^8^ Orthogonal polynomial (linear and quadratic) contrasts were performed to test the effect of phytase levels in the phosphorus-deficient diets.

**Table 7:** Mechanical (structural and material) properties of femora of grower–finisher pigs slaughtered at 110 kg BW.

Treatment ^1^	Stiffness, kN/mm	Yield Force, kN	Breaking Force, kN	Young’s Modulus, GPa	Yield Strain, %	Yield Stress, MPa	Breaking Strain, %	Breaking Stress, MPa
NC ^2^	1.76 ^ab^	4.25	5.04 ^b^	0.74 ^a^	9.58 ^c^	70.0 ^ab^	12.34 ^b^	83.4 ^a^
250 ^3^	1.91 ^ab^	4.18	5.37 ^b^	0.74 ^a^	8.23 ^bc^	58.6 ^a^	15.9 ^b^	74.4 ^a^
500 ^3^	1.92 ^ab^	4.40	4.12 ^a^	0.79 ^ab^	5.83 ^a^	64.5 ^a^	8.51 ^a^	74.1 ^a^
1000 ^3^	2.16 ^b^	4.50	5.22 ^b^	1.45 ^c^	6.77 ^ab^	92.2 ^b^	8.63 ^a^	108.7 ^b^
1500 ^3^	1.67 ^a^	4.35	4.69 ^ab^	1.21 ^bc^	6.91 ^ab^	81.1 ^ab^	9.16 ^a^	89.5 ^ab^
PC ^4^	2.05 ^ab^	4.42	5.30 ^b^	1.28 ^bc^	6.64 ^ab^	80.9 ^ab^	9.29 ^a^	95.9 ^b^
SEM ^5^	0.28	0.44	0.59	352	1.15	15.8	1.77	19.0
*p*-value								
TRT ^6^	0.013	0.630	<0.001	<0.001	<0.001	0.001	<0.001	0.005
PHY ^7^	0.011	0.581	0.002	<0.001	<0.001	0.001	<0.001	0.005
Linear ^8^	0.809	0.280	0.196	<0.001	<0.001	0.002	<0.001	0.034
Quadratic ^8^	0.005	0.637	0.420	0.778	<0.001	0.261	0.562	0.559

^a–c^ Means with different superscripts, within the same column, are statistically different from one another (*p* < 0.05) based on Tukey’s post hoc test. ^1^ There were a total of 6 dietary treatments. Diets 1–5 were phosphorus-deficient diets: with 0 (NC), 250, 500, 1000, or 1500 phytase units FTU/kg (RONOZYME HiPhos phytase, DSM, Poland), respectively. A sixth diet (positive control; PC) was formulated with increased di-calcium phosphate to meet the nutrient requirements of the pigs [15]. ^2^ NC—negative control. ^3^ Phytase dose (FTU/kg feed) added to NC. ^4^ PC—positive control. ^5^ SEM—standard error of the means. ^6^ P-value for overall effect of dietary treatment (Diets 1–6). ^7^ *p*-value for phytase effect in phosphorus-deficient diets (Diets 1–5). ^8^ Orthogonal polynomial (linear and quadratic) contrasts were performed to test the effect of phytase level in the phosphorus-deficient diets.

## References

[1] Humer E., Schwarz C., Schedle K. (2015). Phytate in pig and poultry nutrition. J. Anim. Physiol. Anim. Nutr..

[2] Oster M., Just F., Büsing K., Wolf P., Polley C., Vollmar B., Muráni E., Ponsuksili S., Wimmers K. (2016). Toward improved phosphorus efficiency in monogastrics—Interplay of serum, minerals, bone, and immune system after divergent dietary phosphorus supply in swine. Am. J. Physiol. Regul. Integr. Comp. Physiol..

[3] Iqbal T.H., Lewis K.O., Cooper B.T. (1994). Phytase activity in the human and rat small intestine. Gut.

[4] Walz O.P., Pallauf J. (2002). Microbial phytase combined with amino acid supplementation reduces P and N excretion of growing and finishing pigs without loss of performance. Int. J. Food Sci. Technol..

[5] Woyengo T.A., Nyachoti C.M. (2013). Anti-nutritional effects of phytic acid in diets for pigs and poultry—Current knowledge and directions for future research. Can. J. Anim. Sci..

[6] Kornegay E.T., Qian H. (1996). Replacement of inorganic P by microbial phytase for young pigs fed on a maize-soybean-meal diet. Br. J. Nutr..

[7] El-Hack M.E.A., Alagawany M.E., Arif M., Emam M., Saeed M.M., Arain M.A., Siyal F.A., Patra A., Elnesr S.S., Khan R.U. (2018). The uses of microbial phytase as a feed additive in poultry nutrition—A review. Ann. Anim. Sci..

[8] Augspurger N.R., Webel D.M., Lei X.G., Baker D.H. (2003). Efficacy of an *E. coli* phytase expressed in yeast for releasing phytate-bound phosphorus in young chicks and pigs. J. Anim. Sci..

[9] Adeola O., Olukosi O.A., Jendza J.A., Dilger R.N., Bedford M.R. (2006). Response of growing pigs to *Peniophora lycii*- and *Escherichia coli*-derived phytases or varying ratios of calcium to total phosphorus. Anim. Sci..

[10] Rey C., Combes C., Drouet C., Glimcher M.J. (2009). Bone mineral: Update on chemical composition and structure. Ostoporos. Int..

[11] Tomaszewska E., Muszyński S., Dobrowolski P., Kamiński D., Czech A., Grela E.R., Wiącek D., Tomczyk-Warunek A. (2019). Dried fermented post-extraction rapeseed meal given to sows as an alternative protein source for soybean meal during pregnancy improves bone development of their offspring. Livest. Sci..

[12] Friendship R.M., Wilson M.R., Almond G.W., McMillan I., Hacker R.R., Pieper R., Swaminathan S.S. (1986). Sow wastage: Reasons for and effect on productivity. Can. J. Vet. Res..

[13] Jensen T.B., Kristensen H.H., Toft N. (2012). Quantifying the impact of lameness on welfare and profitability of finisher pigs using expert opinions. Livest. Sci..

[14] EFSA (2012). Scientific opinion on the safety and efficacy of Ronozyme^®^ HiPhos M/L (6-phytase) as a feed additive for poultry and pigs. EFSA J..

[15] NRC (2012). Nutrient Requirements of Swine.

[16] AOAC (2016). Official Methods of Analysis of AOAC International.

[17] Engelen A.J., van der Heeft F.C., Randsdorp P.H., Somers W.A., Schaefer J., van der Vat B.J. (2001). Determination of phytase activity in feed by a colorimetric enzymatic method: Collaborative interlaboratory study. J. AOAC Int..

[18] Suzuki E.Y., Early R.J. (1991). Analysis of chromic oxide in small samples of feeds and feces using chlorine bleach. Can. J. Anim. Sci..

[19] Brestenský M., Nitrayová S., Heger J., Patráš P. (2017). Chromic oxide and acid-insoluble ash as markers in digestibility studies with growing pigs and sows. J. Anim. Physiol. Anim. Nutr..

[20] Adeola O., Lewis A.J., Southern L.L. (2001). Digestion and balance techniques in pigs. Swine Nutrition.

[21] Teixeira A.O., Anderson Corassa A., Moreira L.M., Nogueira E.T., Lopes J.B., Rocha C.M., Ferreira V.P.A. (2016). Bone characteristics of pigs fed different sources of phosphorus. Rev. Colom. Cienc. Pecua.

[22] Santos T.T., Walk C.L., Wilcock P., Cordero G., Chewning J. (2014). Performance and bone characteristics of growing pigs fed diets marginally deficient in available phosphorus and a novel microbial phytase. Can. J. Anim. Sci..

[23] Crenshaw T.D., Peo E.R., Lewis A.J., Moser B.D., Olson D.G. (1981). Influence of age, sex and calcium and phosphorus levels on the mechanical properties of various bones in swine. J. Anim. Sci..

[24] Saraiva A., Donzele J.L., Oliveira R.F.M., Abreu M.L.T., Silva F.C.O., Guimarães S.E.F., Kim S.W. (2012). Phosphorus requirements for 60- to 100-kg pigs selected for high lean deposition under different thermal environments. J. Anim. Sci..

[25] Storskrubb A., Sevón-Aimonen M.L., Uimari P. (2010). Genetic parameters for bone strength, osteochondrosis and meat percentage in Finnish Landrace and Yorkshire pigs. Animal.

[26] Shaw D.T., Rozeboom D.W., Hill G.M., Orth M.W., Rosenstein D.S., Link J.E. (2006). Impact of supplement withdrawal and wheat middling inclusion on bone metabolism, bone strength, and the incidence of bone fractures occurring at slaughter in pigs. J. Anim. Sci..

[27] Pointillart A., Guéguen L. (1993). Meal-feeding and phosphorus ingestion influence calcium bioavailability evaluated by calcium balance and bone breaking strength in pigs. Bone Miner..

[28] Tomaszewska E., Dobrowolski P., Kwiecień M., Winiarska-Mieczan A., Tomczyk A., Muszyński S., Gładyszewska B. (2017). Dose-dependent influence of dietary Cu-glycine complex on bone and hyaline cartilage development in adolescent rats. Ann. Anim. Sci..

[29] Muszyński S., Kwiecień M., Tomaszewska E., Świetlicka I., Dobrowolski P., Kasperek K., Jeżewska-Witkowska G. (2017). Effect of caponization on performance and quality characteristics of long bones in Polbar chickens. Poult. Sci..

[30] Jee W.S.S. (2000). Principles in bone physiology. J. Musculoskel. Neuron. Interact..

[31] Clarke B. (2008). Normal bone anatomy and physiology. Clin. J. Am. Soc. Nephrol..

[32] Kühn I., Schollenberger M., Männer K. (2016). Effect of dietary phytase level on intestinal phytate degradation and bone mineralization in growing pigs. J. Anim. Sci..

[33] Kierończyk B., Rawski M., Józefiak D., Świątkiewicz S. (2017). Infectious and non-infectious factors associated with leg disorders in poultry—A review. Ann. Anim. Sci..

[34] Dersjant-Lia Y., Wealleans A.L., Barnard L.P., Lane S. (2017). Effect of increasing *Buttiauxella* phytase dose on nutrient digestibility and performance in weaned piglets fed corn or wheat based diets. Anim. Feed Sci. Technol..

[35] Schlegel P., Gutzwiller A. (2020). Dietary calcium to digestible phosphorus ratio for optimal growth performance and bone mineralization in growing and finishing pigs. Animals.

[36] Blavi L., Muñoz C.J., Broomhead J.N., Stein H.H. (2019). Effects of a novel corn-expressed E. coli phytase on digestibility of calcium and phosphorous, growth performance, and bone ash in young growing pigs. J. Anim. Sci..

[37] Tsai T.C., Dove R., Bedford M.R., Azain M.J. (2020). Effect of phytase on phosphorous balance in 20-kg barrows fed low or adequate phosphorous diets. Anim. Nutr. J..

[38] Varley P.F., Flynn B., Callan J.J., O’Doherty J.V. (2011). Effect of phytase level in a low phosphorus diet on performance and bone development in weaner pigs and the subsequent effect on finisher pig bone development. Livest. Sci..

[39] Wiśniewska M., Nollet L., Lanckriet A., Vanderbeke E., Petkov S., Outchkourov N., Kasprowicz-Potocka M., Zaworska-Zakrzewska A., Kaczmarek S.A. (2020). Effect of phytase derived from the *E. coli* AppA gene on weaned piglet performance, apparent total tract digestibility and bone mineralization. Animals.

[40] Tomaszewska E., Kwiecień M., Muszyński S., Dobrowolski P., Kasperek K., Blicharski T., Jeżewska-Witkowska G., Grela E.R. (2017). Long-bone properties and development are affected by caponisation and breed in Polish fowls. Br. Poult. Sci..

[41] Tomaszewska E., Kwiecień M., Dobrowolski P., Klebaniuk R., Muszyński S., Olcha M., Blicharski T., Grela E.R. (2018). Dose-dependent effects of probiotic supplementation on bone characteristic and mineralization in female turkeys. Anim. Prod. Sci..

[42] Tomaszewska E., Muszyński S., Dobrowolski P., Kostro K., Jakubczak A., Taszkun I., Żmuda A., Blicharski T., Kędzia P. (2016). Bentonite diminishes DON-induced changes in bone development in mink dams. J. Vet. Res..

[43] Lawlor P.G., Cozannet P., Ryan W.F., Lynch P.B. (2019). Effect of a combination phytase and carbohydrolase enzyme supplement on growth performance and bone mineralization of pigs from six weeks to slaughter at 105 kg. Livest. Sci..

[44] de Faria H.G., Thomaz M.C., dos Santos Ruiz U., Robles-Huaynate R.A., Watanabe P.H., de Melo G.M.P., da Silva S.Z. (2015). Effects of phytase on pig diets digestibilities, bone mineral deposition, performance and manure production. Semina Ciênc. Agrár..

[45] Miesorski M., Gerlinger C., Borgelt L., Lieboldt M.A., Oster M., Wimmers K., Wolf P. Bone mineralization as diagnostic parameter for the assessment of dietary phosphorous supply in pigs—Are there differences between bones?. Proceedings of the 22nd Congress of the European Society of Veterinary and Comparative Nutrition.

[46] Bernau M., Schrott J., Schwanitz S., Kreuzer L.S., Scholz A.M. (2020). “Sex” and body region effects on bone mineralization in male pigs. Arch. Anim. Breed..

[47] Kääntee E. (1983). The significance of the ash content of the third metacarpal bone for the diagnosis of Ca deficiency in the pig. Nord. Vet. Med..

[48] Badoux D.M., Jenkins A. (1974). An introduction to biomechanical principles in primate locomotion and structure. Primate Locomotion.

[49] Rudyk H., Tomaszewska E., Kotsyumbas I., Muszyński S., Tomczyk-Warunek A., Szymańczyk S., Dobrowolski P., Wiącek D., Kamiński D., Brezvyn O. (2019). Bone homeostasis in experimental fumonisins intoxication of rats. Ann. Anim. Sci..

[50] Tomaszewska E., Muszyński S., Dobrowolski P., Wiącek D., Tomczyk-Warunek A., Świetlicka I., Pierzynowski S.G. (2019). Maternal HMB treatment affects bone and hyaline cartilage development in their weaned piglets via the leptin/osteoprotegerin system. J. Anim. Physiol. Anim. Nutr..

[51] Śliwa E. (2010). 2-Oxoglutaric acid administration diminishes fundectomy-induced osteopenia in pigs. J. Anim. Physiol. Anim. Nutr..

[52] Hart N.H., Nimphius S., Rantalainen T., Ireland A., Siafarikas A., Newton R.U. (2017). Mechanical basis of bone strength: Influence of bone material, bone structure and muscle action. J. Musculoskelet. Neuronal Interac..

[53] Ferretti J.L., Capozza R.F., Mondelo N., Zanchetta J.R. (1993). Interrelationships between densitometric, geometric, and mechanical properties of rat femora: Inferences concerning mechanical regulation of bone modeling. J. Bone Miner. Res..

[54] Tomaszewska E., Muszyński S., Dobrowolski P., Kwiecień M., Klebaniuk R., Szymańczyk S., Tomczyk A., Kowalik S., Milczarek A., Świetlicka I. (2018). The influence of dietary replacement of soybean meal with high-tannin faba beans on gut-bone axis and metabolic response in broiler chickens. Ann. Anim. Sci..

[55] van der Meulen M.C.H., Jepsen K.J., Mikić B. (2011). Understanding bone strength: Size isn’t everything. Bone.

[56] Milgrom C., Giladi M., Simkin A., Rand N., Kedem R., Kashtan H., Stein M., Gomori M. (1989). The area moment of inertia of the tibia: A risk factor for stress fractures. J. Biomech..

[57] Flis M., Gugała D., Muszyński S., Dobrowolski P., Kwiecień M., Grela E.R., Tomaszewska E. (2019). The influence of the partial replacing of inorganic salts of calcium, zinc, iron and copper with amino acid complexes on bone development in male pheasants from aviary breeding. Animals.

